# The Decoration of ZnO Nanoparticles by Gamma Aminobutyric Acid, Curcumin Derivative and Silver Nanoparticles: Synthesis, Characterization and Antibacterial Evaluation

**DOI:** 10.3390/nano11020442

**Published:** 2021-02-09

**Authors:** Chanon Talodthaisong, Kittiya Plaeyao, Chatariga Mongseetong, Wissuta Boonta, Oranee Srichaiyapol, Rina Patramanon, Navaphun Kayunkid, Sirinan Kulchat

**Affiliations:** 1 Department of Chemistry, Faculty of Science, Khon Kaen University, Khon Kaen 40002, Thailand; Chanon@kkumail.com (C.T.); kittiya.plaeyao@gmail.com (K.P.); Chatariga.m@kkumail.com (C.M.); bwissuta@kkumail.com (W.B.); 2 Department of Biochemistry, Faculty of Science, Khon Kaen University, Khon Kaen 40002, Thailand; oranee_sr@kkumail.com (O.S.); narin@kku.ac.th (R.P.); 3 College of Nanotechnology, King Mongkut’s Institute of Technology Ladkrabang, Ladkrabang, Bangkok 10520, Thailand; navaphun.ka@kmitl.ac.th

**Keywords:** ZnO nanoparticles, gamma-aminobutyric acid, surface functionalization, antibacterial activity, curcumin derivative

## Abstract

Zinc oxide nanoparticles (ZnO NPs) are applied in various applications in catalysis, biosensing, imaging, and as antibacterial agents. Here we prepared ZnO nanomaterials decorated by *γ*-aminobutyric acid (GABA), curcumin derivatives (CurBF_2_) and silver nanoparticles (CurBF_2_-AgNPs). The structures of all ZnO nanostructures were characterized using Fourier transform infrared (FT-IR) spectroscopy, X-ray diffraction (XRD), UV–VIS spectrophotometry, fluorescence spectrophotometry, scanning electron microscopy (SEM), transmission electron microscopy (TEM), and high-resolution transmission electron microscopy (HR-TEM). Further, their antibacterial activities against Gram-negative (*Escherichia coli*) and Gram-positive (*Staphylococcus aureus*) bacteria were investigated through analysis of minimum inhibitory concentration (MIC) method. Among the prepared nanostructures, the ZnO NPs-GABA/CurBF_2_-AgNPs showed excellent antibacterial activity against both Gram-positive and Gram-negative bacteria. ZnO NPs fabricated here may have potential use in future anti-bacterial compositions and coatings technologies.

## 1. Introduction

Zinc oxide nanoparticles (ZnO NPs) are an important example of inorganic metal oxide NPs that exhibit significant size- and shape-dependent antibacterial activity for a broad range of bacterial species [1,2,3]. ZnO shows better antimicrobial activity when particle size is decreased to the nanometer range (1–100 nm) [4] because nanoscale ZnO can interact with either the surface or the core of bacteria with consequent bactericidal effects [5]. Some studies suggest that the antibacterial function of ZnO may derive from disturbances of the cell membrane [6]. Others suggest that ZnO can, under certain conditions, stimulate the production of reactive oxygen species (ROS), which can destroy bacteria [7]. It has been shown that ZnO can be activated by UV and visible light, leading to ROS, including hydroxide ions (OH^−^), hydrogen peroxide (H_2_O_2_), and oxygen superoxide (O_2_^2−^). The hydroxyl radicals and superoxide can bind to the cell surface, whilst hydrogen peroxide can penetrate bacterial cells and do damage from within [2].

ZnO NPs also have the high surface area, low toxicity, appropriate biocompatibility, and high chemical stability to make them of considerable interest in biomedical studies [8]. ZnO NPs have also been employed for applications such as biosensing [9], catalysis [10,11], photocatalysis [12,13], and imaging [14]. The specific properties required for each application can be obtained by various synthetic methods that control ZnO NP size and morphology. ZnO nanorods, nanospheres, nanotubes, nanowires, and nanoneedles have been obtained by tuning the chemical and physical parameters (solvent, precursors, pH, temperature, etc.) during synthesis [15,16,17]. Different reagents have been employed as capping agents, altering growth kinetics at certain facets and driving morphological diversity. These agents include mercaptoethanol (resulting in spherical particles) [18], 3-aminopropyltriethoxysilane (spherical) [19], monoethanolamine (nanosheet) [20], fatty acids (nanorods) [21], curcumin (semi-spherical core-shell) [8], mercaptoacetic acid (spherical) [22], and enzymes (spherical) [23]. In addition, the incorporation of ZnO NPs and silver nanoparticles was reported previously. Dhara, S. et al. investigated the decoration of AgNPs on ZnO nanoflowers impregnated on eggshell membranes and Eshraghi et al. reported the effect of the incorporation of ZnO and AgNPs on bacterial biofilms. These studies showed that AgNPs decorated on ZnO enhanced the latter’s antimicrobial activity [24,25].

Gamma-amino butyric acid (GABA), is a small organic molecule composed of amino and carboxylic functional groups. It is a non-protein amino acid that is distributed broadly in biological systems [26] and a major inhibitory neurotransmitter in the human central nervous system [27,28]. Due to the non-toxicity of GABA, its simplicity, and its bi-functionality, we utilize it here as a modifying surface agent in the synthesis of ZnO NPs for controlling the size and morphology of the ZnO NPs.

Curcumin can be obtained by the extraction of the rhizomes of *Curcuma longa* and is a yellow-orange dye with low toxicity [29]. It is an active ingredient of turmeric and has been widely evaluated for the treatment of wounds [30], Alzheimer’s disease [31], diabetes [32], inflammatory bowel syndrome [33], and rheumatoid arthritis [34]. Curcumin has carbonyl group functionality and undergoes keto-enol tautomerization. The addition of boron difluoride (BF_2_) to the carbonyl groups of curcumin can inhibit the keto-enol tautomerization, giving CurBF_2_. The latter has vastly different photophysical properties to curcumin, being highly fluorescent, and having markedly higher stability relative to curcumin [35]. Patra et al. [36], reported the functionalization of ZnO nanostructures with curcumin that changed the photophysical properties of the ZnO NPs. The NPs were then applied to sense arsenic ion contamination in water. In this work, we investigate for the first time of the functionalization of ZnO NPs with CurBF_2_ and its resultant properties.

The present work focuses on the preparation of ZnO nanostructures using *γ*-aminobutyric acid (GABA) and a curcumin derivative (CurBF_2_). Moreover, silver nanoparticles stabilized by CurBF_2_ (CurBF_2_-AgNPs) are synthesized and their decoration on ZnO NPs is achieved. Several characterization techniques are used to analyze the structures of these novel nanomaterials as well as their electronic properties. Further, we investigate the antibacterial efficiency of all the as-prepared ZnO nanostructures toward both Gram positive (*S. aureus*) and Gram negative (*E. coli*) bacteria by minimum inhibitory concentration (MIC) procedures. This work is a part of a continuing drive in our research group to synthesize and study new nanomaterials as well as to investigate their antibacterial abilities by natural chemical and nanoscale modifications.

## 2. Materials and Methods

### 2.1. Materials

Zinc chloride anhydrous (ZnCl_2_, 98%) was purchased from Loba Chemie (Mumbai, India). Sodium hydroxide (NaOH, 99%) and methanol (MeOH, 99.9%) were purchased from RCL Labscan (Bangkok, Thailand). Boron trifluoride diethyl etherate (BF_3_O(C_2_H_5_)_2_, 48%) and 1,1′-Carbonyldiimidazole (CDI, C_7_H_6_N_4_O, 97%) were purchased from Acros Organic (Fair Lawn, NJ, USA). *γ*-Amino butyric acid (GABA, C_4_H_9_NO_2_, >99%) was obtained from Sigma-Aldrich (Missouri, MO, USA). Synthetic grade curcumin (C_21_H_20_O_6_, pure > 97%) was purchased from TCI (Tokyo, Japan). Dimethyl sulfoxide (DMSO, C_2_H_6_OS, >95%) and deuterated dimethyl sulfoxide (DMSO-*d*_6_, C_2_D_6_OS, 99.8%) were purchased from Fisher Scientific (Leicestershire, UK) and Eurisotop (Derbyshire, UK), respectively. Ethanol (C_2_H_5_OH, 99.9%) and potassium carbonate (K_2_CO_3_, ≥99.0%) were obtained from Merck (Darmstadt, Germany). Silver nitrate (AgNO_3_, 99.9%) was purchased from POCH™ (Avantor Performance Materials, Sowińskiego, Poland). Deionized (DI) water with specific resistivity of 18.2 MΩ.cm was obtained from a RiO_s_^TM^ Type I Simplicity 185 (Millipore water purification system), ELGA Labwater (Lane End, UK). Bacterial strains (*Staphylococcus aureus* (*S. aureus*) ATCC25923 and *Escherichia coli* (*E. coli*) O157:H7 were purchased from Department of Medical Sciences, Ministry of Public Health (Bangkok, Thailand). 

### 2.2. Methods

#### 2.2.1. Synthesis of CurBF_2_

CurBF_2_ was synthesized by the addition of boron trifluoride diethyl etheate to the enolate unit of curcumin [35]. Briefly, a solution of curcumin (1.5336 g, 4.16 mmol) in methanol 50 mL was added to a 250 mL two-necked round bottom flask equipped with a magnetic bar and a reflux condenser. Next, the solution was heated to 60 °C and then boron trifluoride diethyl etheate (0.77 mL, 6.24 mmol) was added. The color of the solution changed immediately from yellow to dark red. The reaction was stirred and refluxed overnight until a red precipitate formed. The desired product (CurBF_2_) was obtained as a red solid (38.4% yield) after washing with cold methanol and drying under vacuum. ^1^H NMR (Figure S1b) (400 MHz, *d*_6_-DMSO) δ (in ppm): 10.12 (s, 2H), 7.94 (d, J = 15.2 Hz, 2H), 7.49 (s, 2H), 7.36 (d, J = 8.4 Hz, 2H), 7.04 (d, J = 15.2 Hz, 2H), 6.90 (d, J = 8.4 Hz, 2H), 6.47 (s, 1H), 3.87 (s, 6H).

#### 2.2.2. Synthesis of *γ*-Aminobutyric Acid-Capped ZnO NPs (ZnO NPs-GABA)

ZnO NPs were synthesized by a co-precipitation method [8]. Briefly, 2.0584 g of zinc chloride was dissolved in 60 mL of water: ethanol (1:1) solution in a round bottom flask equipped with magnetic stirrer. Then, a solution of GABA (0.4043 g in 1 mL in DI water) was added dropwise to the zinc chloride solution while stirring. The pH of the mixture was adjusted to 11 using 2 M NaOH aqueous solution. Subsequently, this mixture was stirred at room temperature overnight. The solution was then centrifuged at 6000 rpm for 20 min. The resulting precipitate pellet was re-dispersed and washed with a mixture of DI water and ethanol (ratio 1:1) before centrifuging and washing one more time. Finally, the precipitate was dried in an oven at 60 °C for 24 h to obtain 1.1345 g of the desired product (ZnO NPs-GABA). ZnO NPs without GABA surface modification were also synthesized using the same method but without adding GABA.

#### 2.2.3. Functionalization of CurBF_2_ on ZnO NPs-GABA

ZnO NPs-GABA (0.5 g) were dispersed in 15 mL dimethyl sulfoxide in a two-neck round bottom flask. Then, 0.5 g of 1,1′-carbonyldiimidazole (CDI) was added to the DMSO/ZnO NPs-GABA suspension to activate the carboxyl group on the surface of ZnO nanoparticles. Then, after refluxing for 2 h, 0.2 g of CurBF_2_ was added to the solution, changing its color from colorless to blue-purple. The mixture was shaken for 24 h at room temperature. The desired product was purified by centrifugation at 20,000 rpm for 5 min and washed with DI water. The product was dried by evaporating under vacuum and kept in a desiccator for three days to obtain the product (1.0422 g).

#### 2.2.4. The synthesis of Silver Nanoparticles (CurBF_2_-AgNPs) and Their Decoration on ZnO NPs-GABA

The silver nanoparticles were prepared via a modification of a previous report [37]. Briefly, a solution of 20 mM CurBF_2_ in DMSO (750 μL) was added to 68 mL of DI water in a 250 mL round bottom flask. The solution pH was then adjusted to 9 by K_2_CO_3_. Then, the solution was heated to 100 °C and 7.5 mL of 10 mM AgNO_3_ was quickly added to the solution mixture. The mixture was stirred vigorously at 100 °C for 1 h and filtered by micro filter to obtain the CurBF_2_-AgNPs. The concentration of CurBF_2_-AgNPs was estimated to be 59.13 nM based on an extinction coefficient of 1.84 × 10^8^ M^−1^·cm^−1^ at 392 nm for 8 nm diameter citrate-silver nanoparticles [38]. The CurBF_2_-AgNPs decorated on ZnO NPs-GABA were prepared by distribution of ZnO NPs-GABA (0.5 g) in 4 mL of CurBF_2_-AgNPs solution. The solution was sonicated for 10 min to obtain the final product.

#### 2.2.5. Bacteria Strains Preparation

*Escherichia coli* O157:H7 and *Staphylococcus aureus* ATCC25923 were purchased from the Department of Medical Sciences, Ministry of Public Health (Bangkok, Thailand). The bacteria strains were cultured in Mueller Hinton broth (MHB) at 37 °C overnight and then subcultured in 5 mL of the same medium at 37 °C in a 180-rpm shaker-incubator for 3 h to yield a mid-logarithmic growth phase culture.

#### 2.2.6. Antibacterial Screening by Well Diffusion

Antimicrobial tests were performed by the well diffusion method. First, a single colony was grown in Mueller Hinton Broth (MHB) at 37 °C for 24 h. After inoculation for 3 h, the bacteria were diluted in the same media at an inoculum of 1 × 10^6^ CFU/mL. The, bacteria were then swabbed onto three-dimensional MH agar plate. The agar was cut to produce wells of 6 mm diameter. ZnO NPs, Curcumin, CurBF_2_, ZnO NPs-GABA and ZnO NPs GABA-CurBF_2_, ZnO-GABA/CurBF_2_-AgNPs, CurBF_2_-AgNPs and standard drug were dropped into the wells at 30 µL and incubated at 37 °C for 24 h. Gentamicin was used as the positive control and DI water as the negative control. After 24 h of incubation, the inhibition zone was measured on the millimeter (mm) scale.

#### 2.2.7. Minimum Inhibitory Concentrations (MICs) by Broth Microdilution Method

The MICs were determined by the broth microdilution method. Briefly, a range of concentrations of ZnO NPs, CurBF_2_, ZnO NPs-GABA and ZnO NPs GABA-CurBF_2_, ZnO-GABA/CurBF_2_-AgNPs, CurBF_2_-AgNPs and standard drug were prepared by serial dilution of two-fold. The solutions were then added to an equal volume of bacterial suspension (50 μL) in each well of a 96-well plate with the final cell concentration ranging from 10^6^–10^7^ CFU/mL. The plates were incubated at 37 °C for 24 h. The MICs were taken at the lowest concentration of antibacterial agents that inhibits ≥ 99% of the growth of bacteria [39,40].

#### 2.2.8. Characterization Methods

NMR spectra were recorded using a Bruker Avance 400 MHz spectrometer (Bruker, Fällanden, Switzerland) using *d*_6_-DMSO as a solvent. The phase characterization of pristine and functionalized ZnO powder was studied by an X-ray diffractometer (Rigaku, model SmartLab, Tokyo, Japan) with the X-ray source of CuKα radiation (*λ* = 1.5418 nm). The analysis was carried out by a conventional 2theta scan over the range 10–80 degrees. The morphology of ZnO NPs was determined by transmission electron microscope (TEM) and high-resolution transmission electron microscope (HRTEM) using a Thermo Scientific TALOS F200X system (Eindhoven, Netherlands). Fourier transform infrared (FTIR) spectra were recorded with a Perkin Elmer system (spectrum one model, Waltham, MA, USA) using KBr pellets, over the region 400–4000 cm^−1^. The solution mixtures were shaken using a Vision Scientific shaker (VS-201D, Daejeon, Korea). UV-VIS diffuse reflectance spectra were record using UV–VIS diffuse reflectance spectroscopy (Shimadzu UV-3101PC, Tokyo, Japan) over the range of 200–800 nm. UV-VIS spectrum of CurBF_2_-AgNPs was monitored using an Agilent Technologies Cary 60 UV–VIS spectrophotometer (Penang, Malaysia) with 1.0 cm pathlength quartz cells over the range 200–800 nm. Fluorescence spectra were examined by a fluorescence spectrophotometer (Shimazu RF-5301PC, Tokyo, Japan) with slit widths at 5 nm/5 nm using 1.0 cm quartz cells. Particle sizing was determined by dynamic light scattering (DLS), and particle zeta potential by electrophoretic light scattering, using a Malvern Zetasizer Nano series (Nano ZS, Worcestershire, UK) for all samples. Determinations were repeated in triplicate and the data presented as the mean ± standard deviation (SD). All samples for DLS and zeta potential measurements were prepared by dispersing 0.1 g of ZnO NPs in 10 mL DI water and sonicating at room temperature for 10 min before measurement. The morphology and the elemental constitution were investigated by focused ion beam scanning electron microscopes (FIB-SEM, FEI Helios NanoLab G3 CX, Prague, Czech Republic). The elemental composition of AgNPs decorated on ZnO NPs-GABA was investigated by energy dispersive X-ray analysis (EDX) using transmission electron microscopy (Tecnai G2 20 S-Twin, Prague, Czech Republic).

## 3. Results and Discussion

### 3.1. ZnO Nanoparticles Functionalization by γ-Aminobutyric Acid (GABA) and CurBF_2_ and the Decoration of CurBF_2_ on ZnO NPs

**Scheme 1:**
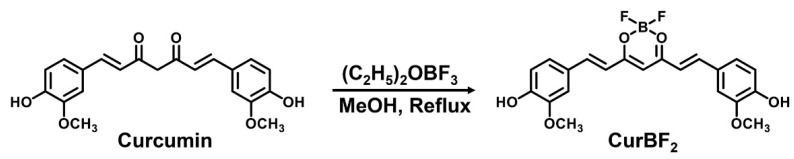
Synthetic scheme of the fluoroboronated curcumin derivative CurBF_2_.

Firstly, we prepared the fluoroboronated curcumin derivative CurBF_2_ as reported in the literature [35]. It was synthesized by the addition of curcumin to boron trifluoride diethyl etherate ((C_2_H_5_)_2_OBF_3_) in refluxing methanol at 60 °C as shown in Scheme 1. The product was obtained as a red solid and characterized by ^1^H-NMR spectroscopy, showing slight downfield shifts of all peaks in the ^1^H-NMR spectrum compared with ^1^H-NMR peaks of pure curcumin (Figure S1a,b).

Next, zinc oxide nanostructures were prepared by a co-precipitation method using ZnCl_2_ and NaOH as the zinc source and the precipitation agent. A solution of ZnCl_2_ was prepared and *γ*-aminobutyric acid (GABA) solution was added dropwise to it. Aqueous NaOH solution was then added to adjust the pH to 11. A proposed reaction mechanism is depicted in Equations (1)–(3). We expected that the surface modification of ZnO NPs with *γ*-aminobutyric acid (GABA) occurred during the ZnO NPs formation to acquire ZnO NPs-GABA.

ZnCl_2_ + 2NaOH → Zn(OH)_2_ + 2NaCl (1)

Zn(OH)_2_ → ZnO + H_2_O (2)

ZnO + GABA → ZnO NPs-GABA(3)

**Scheme 2:**
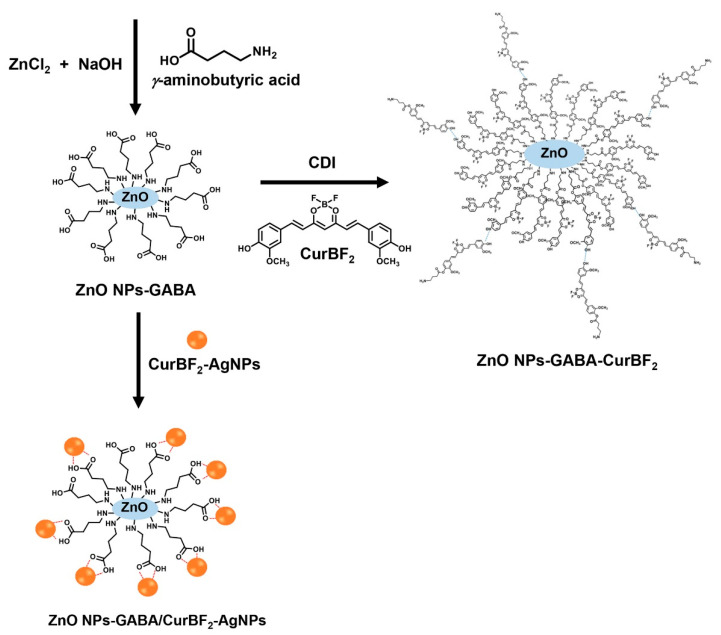
Schematic representation of synthesis of ZnO NPs-GABA, ZnO NPs-GABA-CurBF_2_ and CurBF_2_-AgNPs decorated on ZnO NPs-GABA. GABA binds to the ZnO NP surface via an amine group during synthesis, driving NP growth towards rod-like shapes and leaving carboxylic groups available at the particle surface. Following activation of those carboxylic groups, a fluoroboronated curcumin could be coupled via an ester linkage.

In the second part of this study, we aimed to functionalize the surface of the ZnO NPs-GABA, on which we expected carboxylic groups (R-COOH) of GABA to be available for binding with CurBF_2_. To do this, the activating agent 1,1′-carbonyldiimidazole (CDI) was used to generate reactive carbonyl groups as imidazole carbamate moieties on the surface of ZnO NPs-GABA as depicted in Scheme 2. The hydroxyl groups on the aromatic part of CurBF_2_ can then react with reactive carbonyl, forming an ester linkage. In addition, chains of CurBF_2_ might form at the ZnO NPs-GABA via hydrogen bonding.

**Figure 1:**
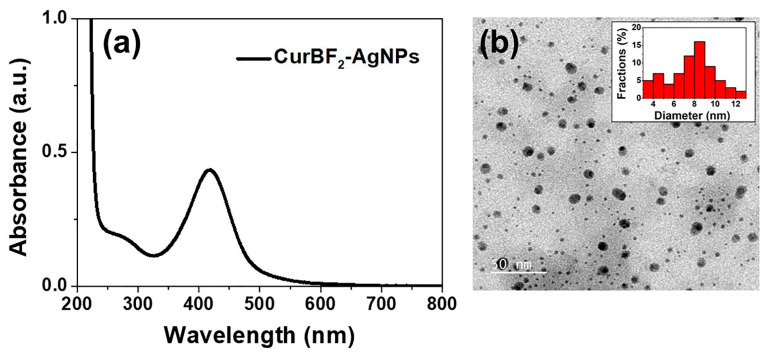
(**a**) UV–VIS spectrum of CurBF_2_-AgNPs, (**b**) TEM image of CurBF_2_-AgNPs, inset the average diameter of 7.75 ± 2.26 nm (*n* = 70).

**Figure 2:**
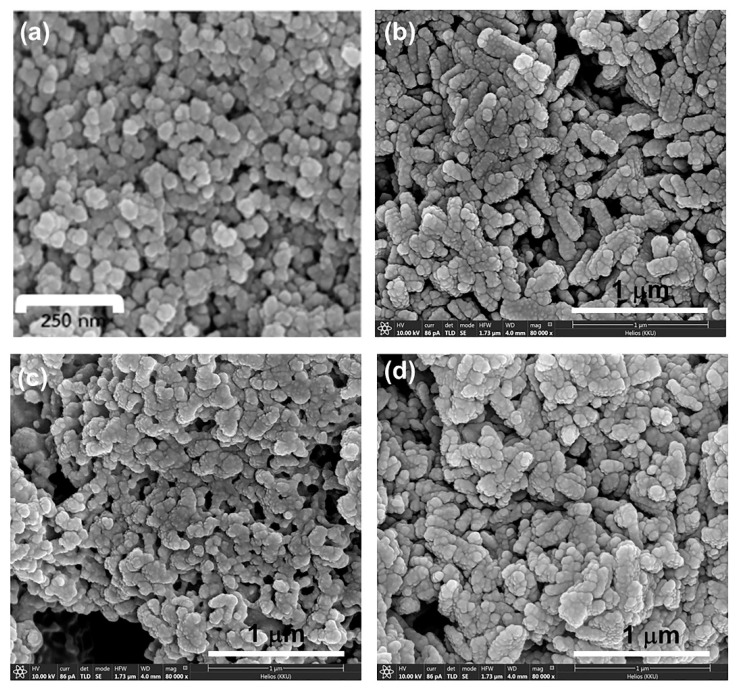
(**a**) SEM image of ZnO NPs, (**b**) FIB-SEM images of ZnO NPs-GABA, (**c**) ZnO NPs-GABA-CurBF_2_, and (**d**) ZnO NPs-GABA/CurBF_2_-AgNPs.

**Figure 3:**
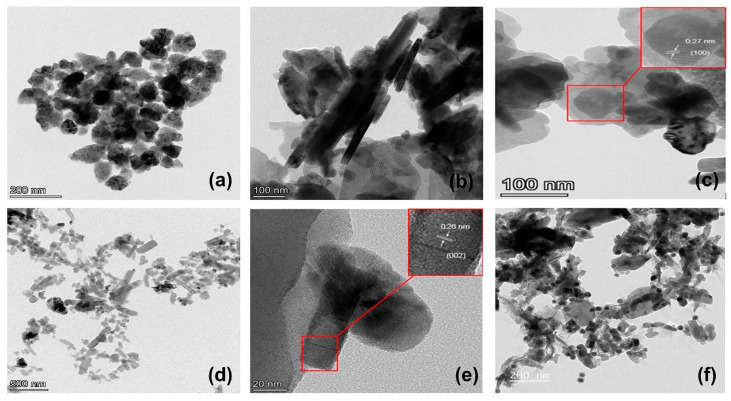
(**a**) TEM image of ZnO NPs, (**b**) TEM and (**c**) HRTEM images of ZnO NPs-GABA, (**d**) TEM and (**e**) HRTEM images of ZnO NPs-GABA-CurBF_2_, and (**f**) TEM image of ZnO NPs-GABA/CurBF_2_-AgNPs.

Lastly, we aim to improve the dispersion of ZnO NPs in water by the decoration of AgNPs on the surface of ZnO NPs (Scheme 2) as well as to improve their antibacterial activity (see antibacterial study section). CurBF_2_-AgNPs was firstly prepared and characterized by UV–VIS spectroscopy as shown in Figure 1a. It illustrates the surface plasmon resonance band at 419 nm. Moreover, the morphology of CurBF_2_-AgNPs was investigated by TEM (Figure 1b) revealing good monodispersity with a size distribution of 7.75 ± 2.26 nm (*n* = 70). Next, CurBF_2_-AgNPs decorated on ZnO NPs-GABA were prepared by the dispersion of ZnO NPs-GABA in CurBF_2_-AgNPs solution. The solution was then homogenized using ultrasonication for 10 min. The morphology of the ZnO NPs-GABA/CurBF_2_-AgNPs from SEM (Figure 2d) indicates the formation of rod-shaped nanomaterials. The TEM images in Figure 3f show that spheroidal particles of CurBF_2_-AgNPs settle on the surface of the ZnO NPs-GABA. The particle size of CurBF_2_-AgNPs decorated on the surface of ZnO NPs-GABA is 22.48 ± 4.15 (*n* = 32), larger than CurBF_2_-AgNPs alone. This could be the aggregation of CurBF_2_-AgNPs upon surface binding. The interaction between ZnO NPs-GABA and CurBF_2_-AgNPs is driven at least in part by electrostatic interactions, with the zeta potentials of the AgNPs and ZnO NPs-GABA being negative and positive, respectively. The interaction drives association of the particles at room temperature and is complete within 10 min of mixing and sonication. The presence of elemental silver in ZnO NPs-GABA/CurBF_2_-AgNPs was confirmed by energy-dispersive X-ray spectroscopy (EDX) as shown in Figure S2. The results indicated a major peak due to elemental silver near 3 keV [41] being 7.1% of total content. In addition, elemental mapping of ZnO NPs-GABA/CurBF_2_-AgNPs (Figure S3) also confirms the presence of zinc, oxygen, nitrogen, boron, fluorine, and silver.

### 3.2. Morphological Analysis of the ZnO Nanostructures

The SEM and TEM images (Figure 2a and Figure 3a) of the ZnO NPs confirm a spheroid morphology with a size distribution of 76.40 ± 16.31 nm (Table 1, Figure S4). The morphology of ZnO NPs-GABA, and ZnO NPs-GABA-CurBF_2_ (Figure 2b,c and Figure 3b,d) show a mixture of sphere and rod-like shapes. This may be due to synthesis at high pH values (pH = 11 in this experiment). Thus, ZnO can directly precipitate and prefers to grow as nanorods [42] due to the surface modification by GABA. The size distribution of the ZnO NPs-GABA spheres is 31.75 ± 10.42 nm, while the length and average diameter of the rod-shaped particles are 139.24 ± 38.72 nm and 31.36 ± 9.13 nm, respectively (Table 1 and Figure S5). Meanwhile, the dimensions of both spheres (53.46 ± 14.60 nm) and rods of ZnO NPs GABA-CurBF_2_ (length and average diameter: 56.54 ± 12.00 nm and 141.44 ± 47.57 nm) are larger than ZnO NPs-GABA (Table 1 and Figure S6). The results indicate that the average particles size of ZnO NPs-GABA was smaller than the size of ZnO NPs, likely because GABA inhibited the growth of ZnO NPs during the co-precipitation process. In addition, HRTEM images of ZnO NPs-GABA and ZnO NPs-GABA-CurBF_2_ (Figure 3c,e) show lattice fringes of *d* = 0.27 nm and 0.26 nm, corresponding to the (100) [43] and (002) [44] interplanar spacing of the wurtzite ZnO phase. The rod-like growth of ZnO NPs-GABA and ZnO NPs-GABA-CurBF_2_ is attributed to the attachment of GABA molecules that encourage the anisotropic growth of ZnO along the (001) direction, leading to nano-rod-like shapes [45]. From the above results, the size of samples determined by TEM are always larger than those determined by XRD. We note that the size extracted from XRD via the Scherrer equation correspond to the average size of the crystalline domain in the particles [46] whereas that determined by TEM is the absolute size of the ZnO NPs. This suggests that at least some particles contain more than one crystalline domain, or that aggregation of ZnO nanocrystallites occurs [47].

### 3.3. Zeta Potential

Next, we evaluated the zeta potential values of the ZnO nanostructures and those decorated with GABA and CurBF_2_-AgNPs. The pH solutions of all samples were determined as shown in Table S1. Wurtzite-type ZnO NPs are recognized to have positive surface charges in the as-prepared state [46]. The result showed that unmodified ZnO NPs showed zeta potential of 5.3 ± 1.1 mV while ZnO NPs modified by GABA demonstrated a zeta potential value of 24.9 ± 0.3 mV. This suggests that GABA was attached to the surface of ZnO NPs. The pH of the ZnO NPs-GABA solution for zeta potential measurements was 7.47. This pH is intermediate between the pKa of the GABA carboxyl and amine groups, and thus the GABA is expected to be neutral. The fact that the zeta potential studies show that ZnO NPs-GABA is in fact positively charged is similar to the result obtained by Kim et al. [46], who functionalized ZnO NPs with L-serine and measured a zeta potential of +26.8 mV at pH 7.0. After ZnO NPs-GABA was covalently attached with CurBF_2_, this value shifted to −9.8 ± 0.5 mV. This may be due to the negative charge of CurBF_2_ influencing the charge on the ZnO NPs’ surface. Moreover, the as-prepared CurBF_2_-AgNPs exhibited zeta potential value of −25.5 ± 1.2 mV indicating high colloid stability [48]. Once CurBF_2_-AgNPs were decorated on the surface of ZnO NPs-GABA, the latter’s zeta potential value changed to −4.5 ± 1.1 mV. This confirms the successful binding of AgNPs to the ZnO NP surface.

### 3.4. Dynamic Light Scattering

DLS was used to determine the hydrodynamic size of all nanomaterial solutions prepared in this work and all results are collected in Table S1. The hydrodynamic sizes of the ZnO NPs (2136 ± 96 nm) were bigger than that of the ZnO NPs-GABA (926 ± 90 nm), corresponding well with the results from TEM. ZnO NPs-GABA-CurBF_2_ have hydrodynamic diameter of 2129 ± 86 nm, consistent with the attachment of CurBF_2_ on the surface of ZnO NPs-GABA. Next, we evaluated the hydrodynamic size of CurBF_2_-AgNPs revealing a value of 40 ± 1 nm, while the hydrodynamic size of the ZnO NPs-GABA decorated with CurBF_2_-AgNPs shows the value 5317 ± 579 nm. This is consistent with the imaging and Zeta potential studies suggesting that the AgNPs are indeed successfully decorated on the surface of ZnO NPs-GABA. The measured hydrodynamic sizes of all nanomaterials are larger than those measured by TEM. This may be due to the DLS experiments being undertaken in aqueous solution, where agglomeration of nanoparticles or the influence of capping molecules on the hydration sphere can be profound [37].

### 3.5. Crystallinity of ZnO Nanostructures

**Figure 4:**
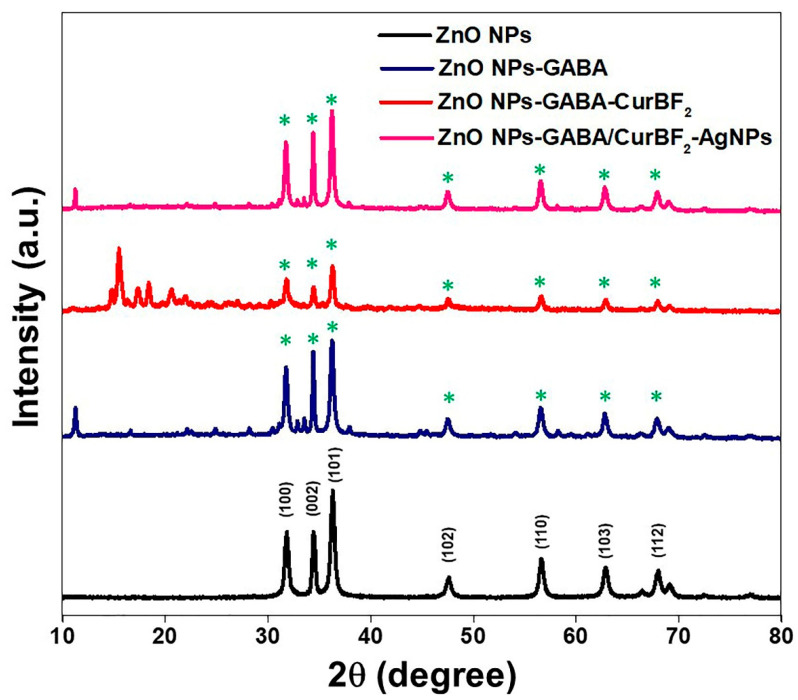
XRD patterns of ZnO NPs, ZnO NPs-GABA, ZnO NPs-GABA-CurBF_2_ and ZnO NPs-GABA/CurBF_2_-AgNPs powder (the * on top of each peak in the ZnO NPs-GABA, ZnO NPs-GABA-CurBF_2_, and ZnO NPs-GABA/CurBF_2_-AgNPs diffractograms are peaks that also appear for ZnO NPs).

XRD analysis was used to confirm the formation of ZnO crystal phase and to investigate the influence of the surface modification on the ZnO NPs. Figure 4 shows the X-Ray diffraction (XRD) patterns of the prepared ZnO NPs, ZnO NPs-GABA, and ZnO NPs-GABA-CurBF_2_, and ZnO NPs-GABA/CurBF_2_-AgNPs. All the ZnO diffraction peaks were found at 2*θ* of 31.82° (*h k l*, 100), 34.43° (*h k l*, 002), 36.39° (*h k l*, 101), 47.65° (*h k l*, 102), 56.62° (*h k l*, 110), 62.91° (*h k l*, 103), 66.52° (*h k l*, 200), 68.13° (*h k l*, 112), 69.11° (*h k l*, 201), and 77.01° (*h k l*, 202). The observed peaks in the diffraction patterns of these as-prepared ZnO NPs are in good agreement with the JCPDS profile of hexagonal wurtzite zinc oxide (JCPDS #36-1451) [8,49]. The patterns of pure GABA at 2*θ* of 10–70° and pure CurBF_2_ at 2*θ* of 10–30°, as shown in Figure S7, are the characteristic peaks of both compounds. The additional peaks of ZnO NPs-GABA found at 16.60°, 22.04°, 24.90°, 28.14°, and 54.02° were assigned to GABA. In addition, the characteristic peaks of CurBF_2_ on ZnO NPs-GABA-CurBF_2_ at 14.76°, 15.51°, 17.32°, 18.38°, 20.60°, and 21.97° were clearly present. These results suggest firstly that the addition of GABA, CurBF_2_, and CurBF_2_-AgNPs to the reactions did not change the crystal structure of the ZnO NP cores. The sharp and intense peaks also indicate good crystallinity of ZnO NPs for all conditions. The Scherrer equation [36] can indicate the average crystallite size of zinc oxide nanoparticle samples via line broadening, estimated by the full width at half maximum (FWHM). By this model, Equation (4) was used to calculate the average crystallite sizes of our as-prepared zinc oxide nanoparticles:

*D*_XRD_ = 0.9 *λβ*/cos *θ* (4)

where *D*_XRD_ is the average crystallite size in nm, *λ* is the X-ray wavelength (*λ* = 1.5418 A), *β* is the FWHM of the highest intensity peak in radians, and *θ* is the diffraction peak half angle. The peak of the (100) plane was used to determine relative crystallite size of each prepared zinc oxide nanoparticle sample. Crystallite sizes of 19.98 nm, 25.41 nm, 26.50 nm, and 26.15 nm were estimated for ZnO NPs, ZnO NPs-GABA, ZnO NPs-GABA-CurBF_2_, and ZnO NPs-GABA/CurBF_2_-AgNPs, respectively. The crystallites of ZnO NPs-GABA being estimated to be larger than those of ZnO NPs may be due to a morphology change from spherical to rod-shaped (Figure 3b). The estimated crystalline sizes of ZnO NPs-GABA and ZnO NPs-GABA-CurBF_2_, ZnO NPs-GABA/CurBF_2_-AgNPs, and their morphology were similar.

### 3.6. Surface Functional Group Analysis

**Figure 5:**
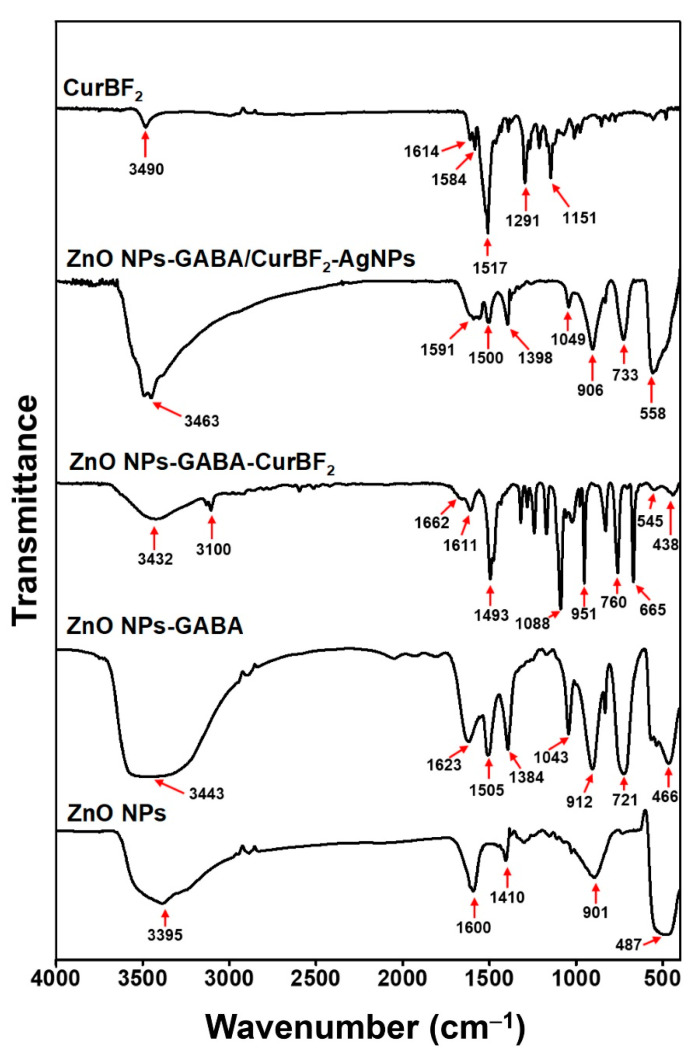
FTIR spectra of CurBF_2_, ZnO NPs-GABA/CurBF_2_-AgNPs, ZnO NPs-GABA-CurBF_2_, ZnO NPs-GABA, and ZnO NPs.

The functional groups on the surface of ZnO NPs were investigated using FTIR spectroscopy. The FTIR spectra of as-prepared ZnO NPs along with free CurBF_2_ are shown in Figure 5. FTIR spectra of ZnO NPs clearly show a broad peak between 3000 and 3600 cm^−1^ attributed to O-H stretching vibration of the hydroxyl groups on the surface of ZnO [8,50]. The peak at 487 cm^−1^ in ZnO is shifted to 466 cm^−1^ in the spectrum of ZnO NPs-GABA, the latter also showing the characteristic peak of Zn-O bonds at 466 cm^−1^. The peak shift from 487 cm^−1^ to 466 cm^−1^ suggests the presence of altered bonding, attributed to the interaction with GABA at the NP surface. The peaks for ZnO NPs-GABA at 1623 cm^−1^ are assigned to stretching vibrations of carbonyl groups (C=O group) while the peak at 1505 cm^−1^ is characteristic of C-N bending, both indicating the presence of γ-aminobutyric acid on the surface of modified ZnO NPs. The broad peak between 3000 and 3600 cm^−1^ and the peak at 438 cm^−1^ in the spectrum of ZnO NPs-GABA-CurBF_2_ imply the presence of hydroxyl groups and Zn-O bonds, respectively. The peak at 1662 cm^−1^ may be due to carbonyl asymmetric vibration in the enol form of CurBF_2_ [36]. The peaks at 1493 cm^−1^, 1088 cm^−1^, 951 cm^−1^, 760 cm^−1^, and 655 cm^−1^ in the spectrum of ZnO NPs-GABA-CurBF_2_ are the characteristic peaks of C-H bending, C-O stretching, C=C bending, indicating that CurBF_2_ was successfully attached at the surface of ZnO NPs-GABA. The IR spectrum of ZnO NPs-GABA/CurBF_2_-AgNPs showed peaks characteristic of ZnO NPs-GABA. These peaks include the peaks of *γ*-aminobutyric acid at 1591 cm^−1^ and 1505 cm^−1^, assigned to the C=O stretch and C-N bending vibration, respectively. However, the intensity of these peaks decreased for ZnO NPs-GABA/CurBF_2_-AgNPs due to the binding of CurBF_2_-AgNPs to the ZnO NPs-GABA surface [51].

### 3.7. The Photophysical Properties of ZnO Nanostructures

#### 3.7.1. UV–VIS Diffuse Reflectance Spectra

**Figure 6:**
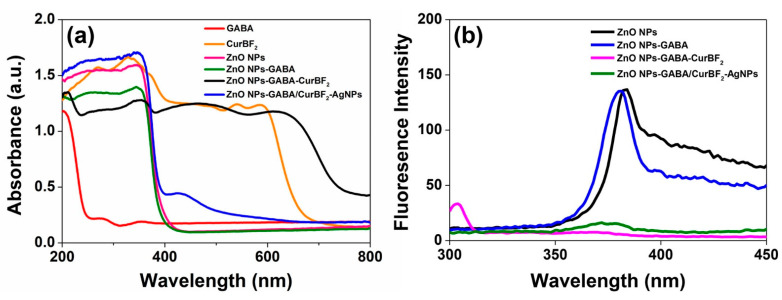
(**a**) UV–VIS diffuse reflectance spectra of free GABA and CurBF_2_, and powders of ZnO NPs, ZnO NPs-GABA, ZnO NPs-GABA-CurBF_2_, and ZnO NPs-GABA/CurBF_2_-AgNPs at room temperature. (**b**) Fluorescence spectra of ZnO NPs, ZnO NPs-GABA, ZnO NPs-GABA-CurBF_2_, and ZnO NPs-GABA/CurBF_2_-AgNPs with slit widths at 5 nm/5 nm excited at 260 nm.

Figure 6a depicts the UV–VIS diffuse reflectance spectra of powders of the as-prepared ZnO NPs, ZnO NPs-GABA, ZnO NPs-GABA-CurBF_2_, ZnO NPs-GABA/CurBF_2_-AgNPs, and free GABA and CurBF_2_. The spectra of all nanostructures show a similar broad absorption spectrum with a maximum at about 340 nm and an onset at about 400 nm, which are characteristic ZnO as a wide band gap semiconductor. For ZnO NPs-GABA-CurBF_2_, other strong absorption maxima were observed at around 220 nm, 480 nm, and 610 nm with absorption peak onsets at about 250 nm, 570 nm, and 680 nm, indicating the presence of both GABA and CurBF_2_ on the surface of the ZnO NPs. The position of GABA and CurBF_2_ peaks in ZnO NPs-GABA-CurBF_2_ is, however, redshifted compared to free GABA and CurBF_2_. This shift may be due to the interaction of GABA and CurBF_2_ with zinc ions that reduces the band gap between π–π* electronic transition of CurBF_2_ [36]. In addition, the absorption band of ZnO NPs-GABA/CurBF_2_-AgNPs occurs at 426 nm with an onset of 500 nm, further evidence of successful decoration of AgNPs on the surface of ZnO NPs-GABA.

#### 3.7.2. Fluorescence Spectra

The photoluminescence (PL) spectra of ZnO NPs, ZnO NPs-GABA, ZnO NPs-GABA-CurBF_2_ and ZnO NPs-GABA/CurBF_2_-AgNPs with excitation at 260 nm were also investigated in order to determine the influence of capping molecules/NPs. The results shown in Figure 6b indicate that only ZnO NPs and ZnO NPs-GABA exhibit an emission band at about 380 nm. This blue-violet emission band was also reported by Sengupta et al. and Mahmoud et al. [52,53] and is attributed to recombination of photo-generated excitons. After the modification of ZnO NPs-GABA with CurBF_2_ and combining with CurBF_2_-AgNPs, those emission bands were quenched, likely by charge transfer to the electron-deficient CurBF_2_. Since plenty of electron-rich hydroxy groups are present on the native surface of ZnO NPs, the CurBF_2_ may possibly interact with these electron-rich hydroxyl groups, becoming efficient surface traps for electrons [54].

### 3.8. Antibacterial Activity

#### 3.8.1. Inhibition Zone Method

**Figure 7:**
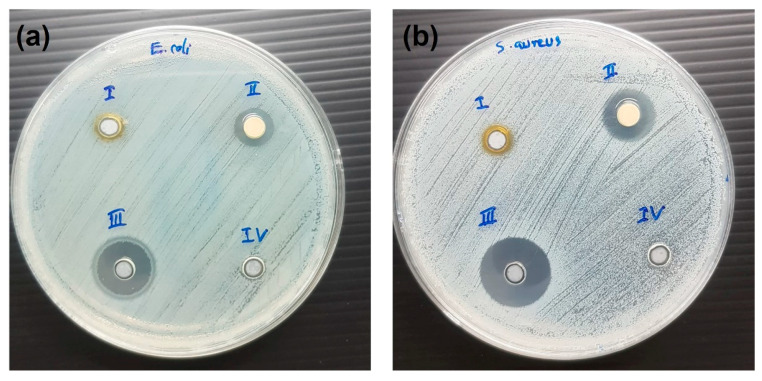
Antimicrobial activity of (i) CurBF*_2_*-AgNPs (ii) ZnO-GABA/CurBF_2_-AgNPs, (iii) gentamicin, (iv) DI water against (**a**) *E. coli* and (**b**) *S. aureus*.

Antibacterial activities by the well-diffusion method for ZnO NPs, ZnO NPs-GABA and ZnO NPs-GABA-CurBF_2_ are shown in Figure S8. The inhibition zones of all samples were absent due to the poor solubility and poor dispersion of ZnO NPs in water leading to impermeability. However, for ZnO NPs-GABA/CurBF_2_-AgNPs enhanced their dispersivity in aqueous phase allowed clear inhibition zones and anti-bacterial activity to be observed (see Figure 7). ZnO NPs-GABA/CurBF_2_-AgNPs exhibit strong antimicrobial activity against both *E. coli* and *S. aureus* bacteria, forming zones of inhibition of diameters 10.17 ± 0.24 and 13.50 ± 0.41 mm, respectively, as shown in Table 2. In comparison, CurBF_2_-AgNPs showed antibacterial activity against only *S. aureus* with inhibition zone of 7.33 ± 0.29 mm (Table 2). These results demonstrate that the combination of ZnO NPs with CurBF_2_-AgNPs gives a synergistic antibacterial effect [25] as compared to AgNPs or ZnO NPs alone.

#### 3.8.2. Minimum Inhibitory Concentration (MIC) Determination

We then have investigated the MIC values of ZnO-NPs, ZnO NPs-GABA, ZnO NPs-GABA-CurBF_2_, and ZnO NPs-GABA/CurBF_2_-AgNPs for their antibacterial activities against Gram positive (*S. aureus*) and Gram negative (*E. coli*) bacteria as shown in Table 3. The MIC of ZnO NPs against *S. aureus* was found to be 100 μg/mL whilst an inhibition effect of these NPs towards *E. coli* was not found. Also, a bacterial inhibition of ZnO NPs-GABA-CurBF₂ against *E. coli* was not found, whilst MIC of ZnO NPs-GABA-CurBF₂ against *S. aureus* were found to be 200 μg/mL. It is clear that ZnO NPs and ZnO NPs-GABA-CurBF₂ exhibited a potent inhibition effect against Gram-positive bacteria. For ZnO NPs-GABA, MIC values against *S. aureus* and *E. coli* were observed to be 100 μg/mL and 200 μg/mL, respectively. Therefore, the synthesized ZnO NPs-GABA can be an effective nano-agent against both Gram-negative and Gram-positive bacterial cells. Conversely, treating both Grams of bacteria with GABA and CurBF_2_ showed no antibacterial effect (Table S2). Interestingly, the antimicrobial activity of ZnO NPs-GABA/CurBF_2_-AgNPs is enhanced against both *S. aureus* and *E. coli* with lower MIC values than the other ZnO NPs which are ≤6.25 µg/mL and 12.5 µg/mL, respectively. Therefore, we can confirm that AgNPs decorated ZnO NPs-GABA nanocomposite can improve the antimicrobial activity of ZnO NPs with synergistic effect.

These antibacterial activity studies indicate that the bactericidal effects of all the tested agents were much more noticeable against *S. aureus* than against *E. coli*. This difference is likely due to the different structure of the cell membrane, together with the cell wall composition of Gram-positive and Gram-negative bacteria. It could also depend on the differences of cell envelope components. The cell wall of Gram-negative bacteria (*E. coli*) is wavy and double-layered, with the presence of an outer membrane with high amounts of lipopolysaccharides. On the other hand, Gram-positive bacteria (*S. aureus*) feature a smooth and single-layered cell wall and the absence of an outer membrane, as well as virtually no lipopolysaccharides. These differences may account for the greater susceptibility of Gram-positive bacteria to the antibacterial agents tested here [55]. Moreover, the sizes of ZnO NPs also play a significant role in antibacterial activities. ZnO NPs with smaller sizes can easily penetrate bacterial cell membranes, thus increasing their antibacterial activity [56,57]. In this work, ZnO NPs-GABA demonstrated the smallest size with the highest specific surface areas compared to other as-prepared ZnO NPs, and also showed the highest efficiency of antibacterial activity. Furthermore, ZnO NPs-GABA/CurBF_2_-AgNPs illustrated an excellent antibacterial efficiency due to a synergistic effect and better dispersion in aqueous phases.

## 4. Conclusions

Zinc oxide nanoparticles (ZnO NPs) were prepared by a simple method with a surface modification with gamma-amino butyric acid (GABA) and were then covalently attached with CurBF_2_. The nanostructures of ZnO NPs were observed to be spherical and rod-like shapes with a hexagonal crystal structure. Modifying ZnO NPs-GABA surface with CurBF_2_ (ZnO NPs-GABA-CurBF_2_) resulted in enhanced near-IR absorption. Finally, as-prepared ZnO NPs showed antibacterial activities against only Gram-positive (*S. aureus*) for ZnO NPs and ZnO NPs-GABA-CurBF_2_. Meanwhile, ZnO NPs-GABA showed good efficiency of antibacterial activity again both Gram-positive (*S. aureus*) and Gram-negative (*E. coli*) bacteria. In addition, the incorporation of CurBF_2_-AgNPs on the surface of ZnO NPs-GABA showed excellent antibacterial activity with synergistic effect. Thus, these nanomaterials may further have potential for biomedical applications, especially given the facile and inexpensive fabrication method developed here.

## Figures and Tables

**Table 1:** Size of ZnO NPs, ZnO NPs-GABA, and ZnO NPs-GABA-CurBF_2_ from the TEM technique.

Nanoparticles	Spheres (nm)	Rods	
		Diameter (nm)	Length (nm)
ZnO NPs	76.40 ± 16.31	-	-
ZnO NPs-GABA	31.75 ± 10.42	31.36 ± 9.13	139.24 ± 38.72
ZnO NPs-GABA-CurBF_2_	53.46 ± 14.60	56.54 ± 12.00	141.44 ± 47.57

**Table 2:** Zone of inhibition in millimeters of ZnO NPs-GABA/CurBF_2_-AgNPs, CurBF_2_-AgNPs, and standard drug against Gram-negative and Gram-positive bacteria.

Bacteria	Zone of Inhibition (mm)		
	ZnO NPs-GABA/CurBF_2_-AgNPs	CurBF_2_-AgNPs	Gentamicin
*E. coli*	10.17 ± 0.24	0.00 ± 0.00	14.00 ± 1.00
*S. aureus*	13.50 ± 0.41	7.33 ± 0.29	18.00 ± 0.50

Mean value ± SD, the means for triplicate samples. Inhibition zone includes diameter of well (6 mm).

**Table 3:** Minimum inhibitory concentration (MIC) of each agent against Gram-positive and Gram-negative bacteria.

Agents	Gram-Positive Bacteria	Gram-Negative Bacteria
	*S. aureus*	*E. coli*
	MIC (µg/mL)	MIC (µg/mL)
ZnO NPs	100	n.d.
ZnO NPs-GABA	100	200
ZnO NPs-GABA-CurBF₂	200	n.d.
ZnO NPs-GABA/CurBF_2_-AgNPs	≤6.25	12.5
Gentamicin	4	4

n.d., not detectable; Gentamicin was used as a standard antibacterial agent; the MIC is the lowest concentration of agent that inhibited bacterial growth by >99%.

## Data Availability

The research data supporting this publication are given within this paper and as supplementary material.

## Supplementary Materials

The following are available online at https://www.mdpi.com/2079-4991/11/2/442/s1, Figure S1. ^1^H-NMR spectra of (a) Curcumin and (b) CurBF_2_; Figure S2. EDX spectrum of ZnO NPs-GABA/CurBF_2_-AgNPs; Figure S3. (a) EDS image of ZnO NPs-GABA/CurBF_2_-AgNPs and element mapping of (b) Zn, (c) O, (d) N, (e) B, (f) F, and (g) Ag; Figure S4. Histogram showing the diameter distribution of the sphere shape of ZnO NPs with the average diameter of 76.40 ± 16.31 nm (*n* = 50); Figure S5. (a) A histogram showing diameter distribution of the sphere shape of ZnO NPs-GABA with the average diameter of 31.75 ± 10.42 nm (*n* = 50); (b) a histogram showing the distribution of the rod shape of ZnO NPs-GABA with the average diameter of 31.36 ± 9.13 nm (*n* = 50); and (c) a histogram showing the distribution of the rod shape of ZnO-NPs-GABA with the average length of 139.24 ± 38.72 nm (*n* = 50); Figure S6. (a) A histogram showing thre diameter distribution of the sphere shape of ZnO NPs-GABA-CurBF_2_ with the average diameter of 53.46 ± 14.60 nm (*n* = 50); (b) a histogram showing the distribution of the rod shape of ZnO NPs-GABA-CurBF_2_ with the average diameter of 56.54 ± 12.00 nm (*n* = 50); and (c) a histogram showing the distribution of the rod shape of ZnO-NPs-GABA-Cur-BF_2_ with the average length of 141.44 ± 47.57 nm (*n* = 50); Figure S7. XRD patterns of ZnO NPs, ZnO NPs-GABA, ZnO NPs-GABA-CurBF_2_, free *γ*-aminobutyric acid (GABA), and free CurBF_2_ powder (the * on top of each peak in the ZnO NPs-GABA and ZnO NPs-GABA-CurBF_2_ diffractograms are peaks that also appear for ZnO NPs).; Figure S8. Antibacterial activity by well diffusion method: (a) *S. aureus*, position (i) gentamicin, (ii) ZnO NPs-GABA, (iii) ZnO NPs-GABA-CurBF_2_, (iv) DI water. (b) *S. aureus*, position (i) gentamicin, (ii) ZnO NPs, (iii) curcumin, (iv) CurBF_2_, (v) DI water. (c) *E. coli*, position (i) gentamicin, (ii) DI water, (iii) ZnO NPs-GABA, (iv) ZnO NPs-GABA-CurBF_2_. (d) *E. coli*, position (i) gentamicin, (ii) DI water, (iii) ZnO NPs, (iv) curcumin, (v) CurBF_2_; Table S1. pH solution, zeta potential and their hydrodynamic sizes of as-prepared nanomaterials; Table S2. Minimum inhibitory concentration (MIC) of each agent against Gram-positive and Gram-negative bacteria.
