# Supplementary material for: The Decoration of ZnO Nanoparticles by Gamma Aminobutyric Acid, Curcumin Derivative and Silver Nanoparticles: Synthesis, Characterization and Antibacterial Evaluation
Source: Nanomaterials (Basel). 2021 Feb 9;11(2):442. doi: 10.3390/nano11020442 (PMC7916182; doi:10.3390/nano11020442)
Supplement: Supplementary file 1 [file nanomaterials-11-00442-s001.pdf]

nanomaterials

Supplementary Materials

The Decoration of ZnO Nanoparticles by Gamma Aminobutyric Acid, Curcumin Derivative, and Silver Nanoparticles: Synthesis, Characterization, and Antibacterial Evaluation

Chanon Talodthaisong [1], Kittiya Plaeyao [1], Chatariga Mongseetong [1], Wissuta Boonta [1], Oranee Srichaiyapol [2], Rina Patramanon [2], Navaphun Kayunkid [3] and Sirinan Kulchat [1,*]

[1] Department of Chemistry, Faculty of Science, Khon Kaen University, Khon Kaen 40002, Thailand; Chanon@kkumail.com (C.T.); kittiya.plaeyao@gmail.com (K.P.); Chatariga.m@kkumail.com (C.M.); bwissuta@kkumail.com (W.B.)
[2] Department of Biochemistry, Faculty of Science, Khon Kaen University, Khon Kaen 40002, Thailand; oranee_sr@kkumail.com (O.S.); narin@kku.ac.th (R.P.)
[3] College of Nanotechnology, King Mongkut's Institute of Technology Ladkrabang, Ladkrabang, Bangkok 10520, Thailand; navaphun.ka@kmitl.ac.th
* Correspondence: sirikul@kku.ac.th

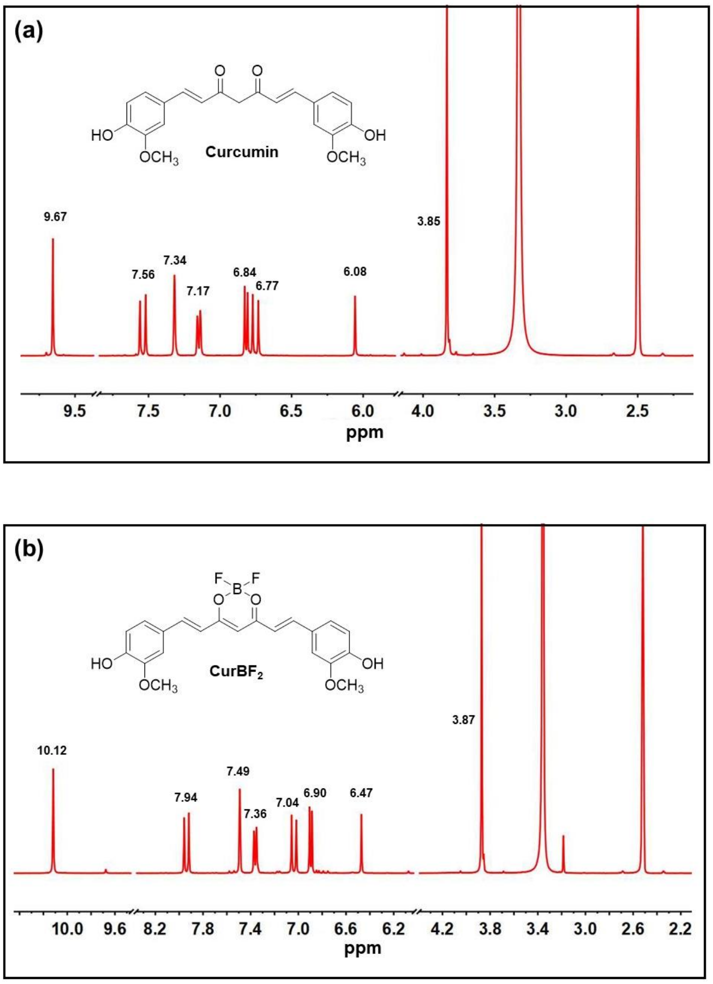

Figure S1. ^{1}H-NMR spectra of: (**a**) Curcumin and (**b**) $CurBF_2$.

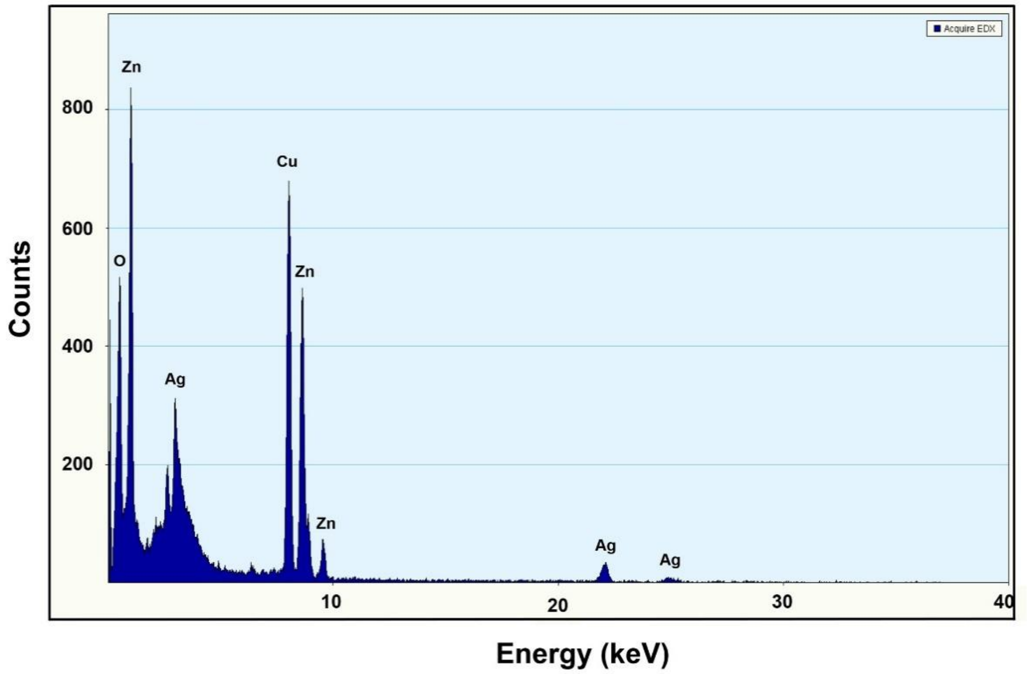

Figure S2. EDX spectrum of ZnO NPs-GABA/CurBF$_2$-AgNPs.

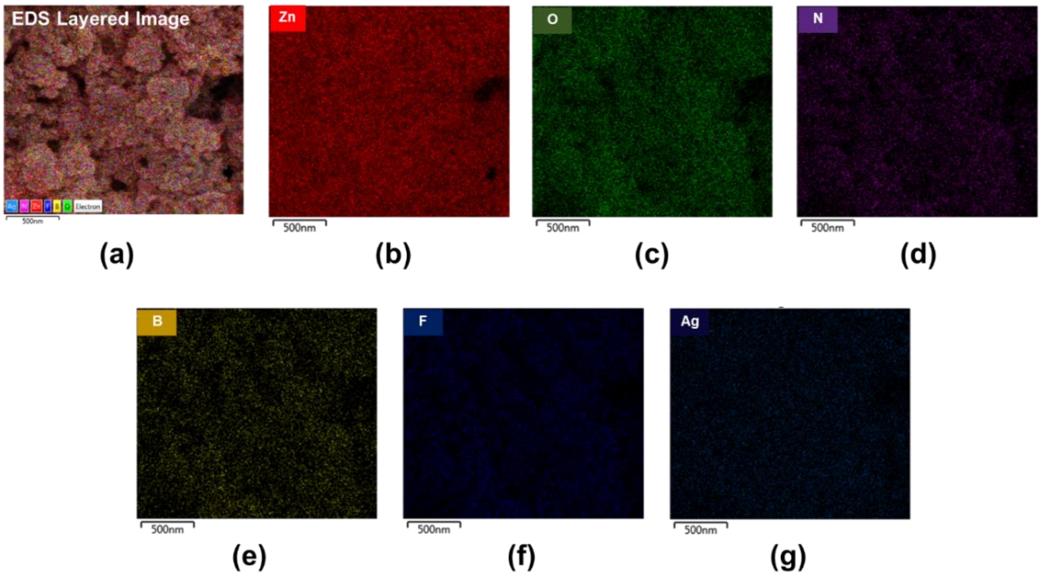

Figure S3. (**a**) EDS image of ZnO NPs-GABA/CurBF$_2$-AgNPs and element mapping of: (**b**) Zn; (**c**) O; (**d**) N; (**e**) B; (**f**) F; (**g**) Ag.

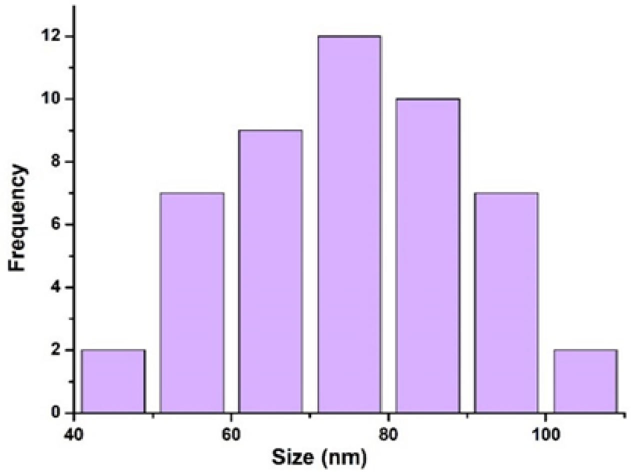

Figure S4. Histogram showing diameter distribution of the sphere shape of ZnO NPs with the average diameter of 76.40 ± 16.31 nm (n = 50).

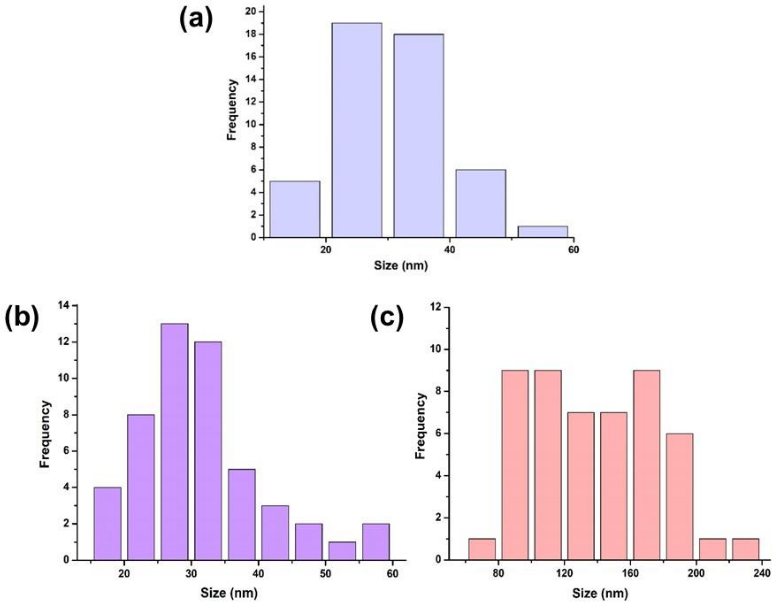

Figure S5. (**a**) A histogram showing diameter distribution of the sphere shape of ZnO NPs-GABA with the average diameter of 31.75 ± 10.42 nm (n = 50); (**b**) a histogram showing the distribution of the rod shape of ZnO NPs-GABA with the average diameter of 31.36 ± 9.13 nm (n = 50); (**c**) a histogram showing the distribution of the rod shape of ZnO-NPs-GABA with the average length of 139.24 ± 38.72 nm (n = 50).

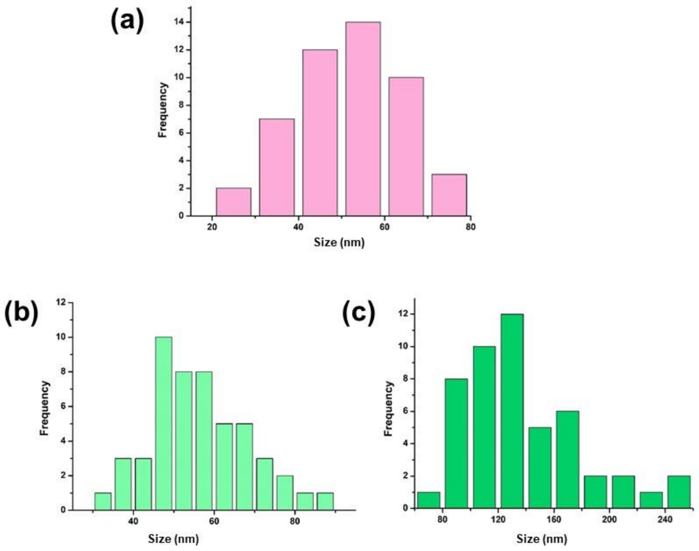

Figure S6. (**a**) A histogram showing diameter distribution of the sphere shape of ZnO NPs-GABA-$CurBF_2$ with the average diameter of 53.46 ± 14.60 nm (*n* = 50); (**b**) a histogram showing the distribution of the rod shape of ZnO NPs-GABA-$CurBF_2$ with the average diameter of 56.54 ± 12.00 nm (*n* = 50); (**c**) a histogram showing the distribution of the rod shape of ZnO-NPs-GABA-$CurBF_2$ with the average length of 141.44 ± 47.57 nm (*n* = 50).

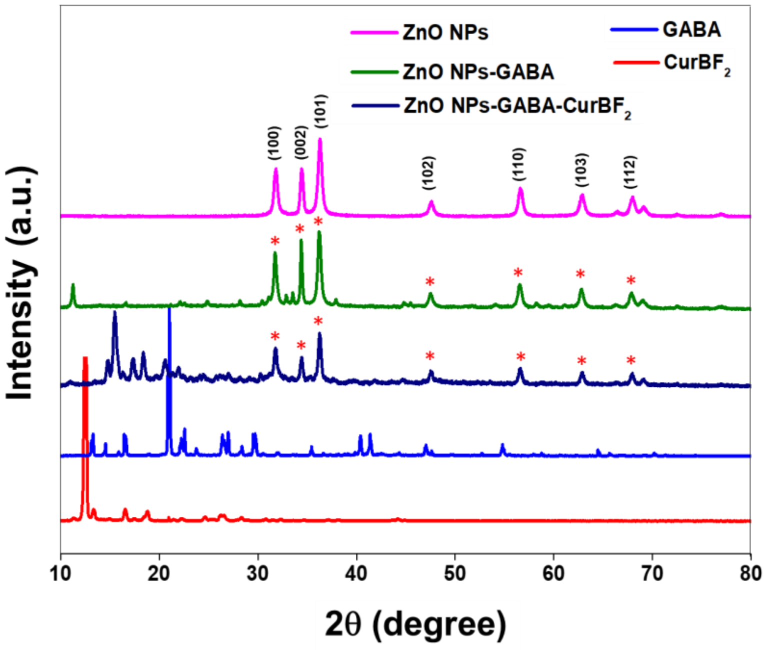

Figure S7. XRD patterns of ZnO NPs, ZnO NPs-GABA, ZnO NPs-GABA-$CurBF_2$, free γ-aminobutyric acid (GABA), and free $CurBF_2$ powder (the * on top of each peak in the ZnO NPs-GABA and ZnO NPs-GABA-$CurBF_2$ diffractograms are peaks that also appear for ZnO NPs).

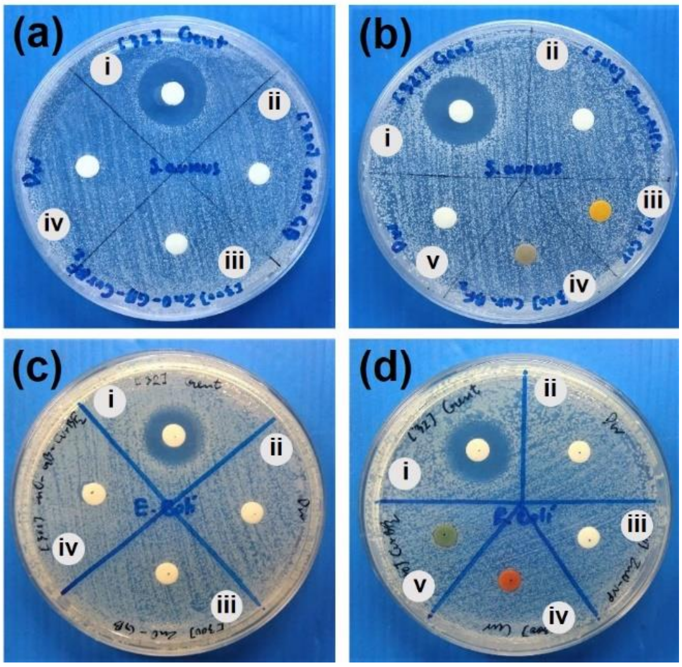

Figure S8. Antibacterial activity by well diffusion method: (**a**) *S. aureus*, position (i) gentamicin, (ii) ZnO NPs-GABA, (iii) ZnO NPs-GABA-CurBF$_2$, (iv) DI water; (**b**) *S. aureus*, position (i) gentamicin, (ii) ZnO NPs, (iii) curcumin, (iv) CurBF$_2$, (v) DI water; (**c**) *E. coli*, position (i) gentamicin, (ii) DI water, (iii) ZnO NPs-GABA, (iv) ZnO NPs-GABA-CurBF$_2$; (**d**) *E. coli*, position (i) gentamicin, (ii) DI water, (iii) ZnO NPs, (iv) curcumin, (v) CurBF$_2$.

Table S1. pH solution, zeta potential and their hydrodynamic sizes of as-prepared nanomaterials.

Nanomaterials	pH of Solution	Zeta Potential (mV)	Diameter (nm)	PDI
ZnO NPs	7.90	5.3 ± 1.1	2136 ± 96	0.55
ZnO NPs-GABA	7.47	24.9 ± 0.3	926 ± 90	0.55
ZnO NPs-GABA-CurBF$_2$	7.83	−9.8 ± 0.5	2129 ± 86	0.25
ZnO NPs-GABA/CurBF$_2$-AgNPs	7.45	−4.5 ± 1.1	5317 ± 579	0.24
CurBF$_2$-AgNPs	8.11	−25.5 ± 1.2	40 ± 1	0.07

Table S2. Minimum inhibitory concentration (MIC) of each agent against Gram-positive and Gram-negative bacteria.

Agents	Gram-Positive Bacteria	Gram-Negative Bacteria
	S. aureus	*E. coli*
	**MIC** (μg/mL)	**MIC** (μg/mL)
CurBF$_2$	n.d.	n.d.
GABA	n.d.	n.d.
3% DMSO	n.d.	n.d.

n.d., not detectable; 3% DMSO was used as a control for the effect of solvent only; the MIC is the lowest concentration of agent that inhibited bacterial growth by >99%.
